# Supplementary material for: Solvent Etching Process for Graphitic Carbon Nitride Photocatalysts Containing Platinum Cocatalyst: Effects of Water Hydrolysis on Photocatalytic Properties and Hydrogen Evolution Behaviors
Source: Nanomaterials (Basel). 2022 Apr 2;12(7):1188. doi: 10.3390/nano12071188 (PMC9000727; doi:10.3390/nano12071188)
Supplement: Supplementary file 1 [file nanomaterials-12-01188-s001.zip › nanomaterials-1650136-supplementary.pdf]

Supplementary Materials

Solvent Etching Process for Graphitic Carbon Nitride Photocatalysts Containing Platinum Cocatalyst: Effects of Water Hydrolysis on Photocatalytic Properties and Hydrogen Evolution Behaviors

Thi Van Anh Hoang, Thi Kim Anh Nguyen, Duc Quang Dao, Phuong Anh Nguyen, Dong Hwi Jeong and Eun Woo Shin *

School of Chemical Engineering, University of Ulsan, Daehakro 93, Nam-gu, Ulsan 44610, Korea;
hoangvananh2203@gmail.com (T.V.A.H.); nguyenthikimanhtb@gmail.com (T.K.A.N.); ddquang221@gmail.com (D.Q.D.); anhphuong.nguyen1150@gmail.com (P.A.N.); jdonghwi@ulsan.ac.kr (D.H.J.)
* Correspondence: ewshin@ulsan.ac.kr; Tel.: +82-52-259-2253

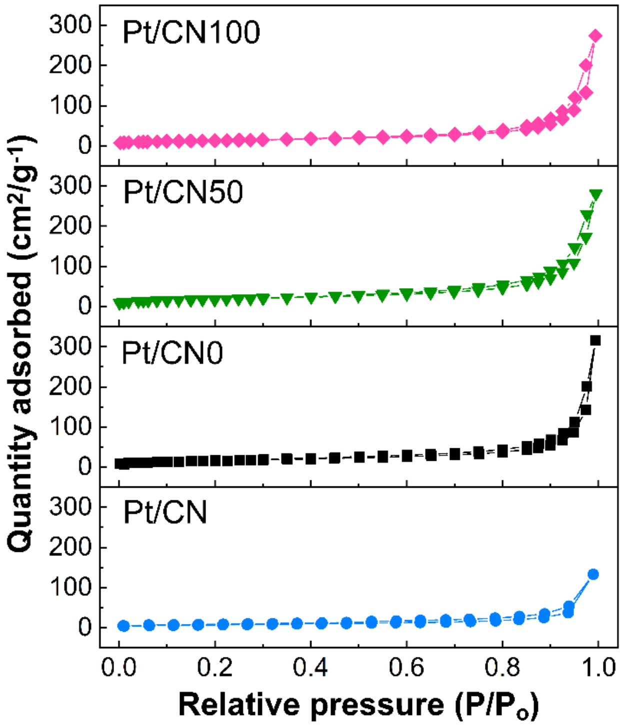

Figure S1. N_2 adsorption-desorption isotherms of the prepared catalysts.

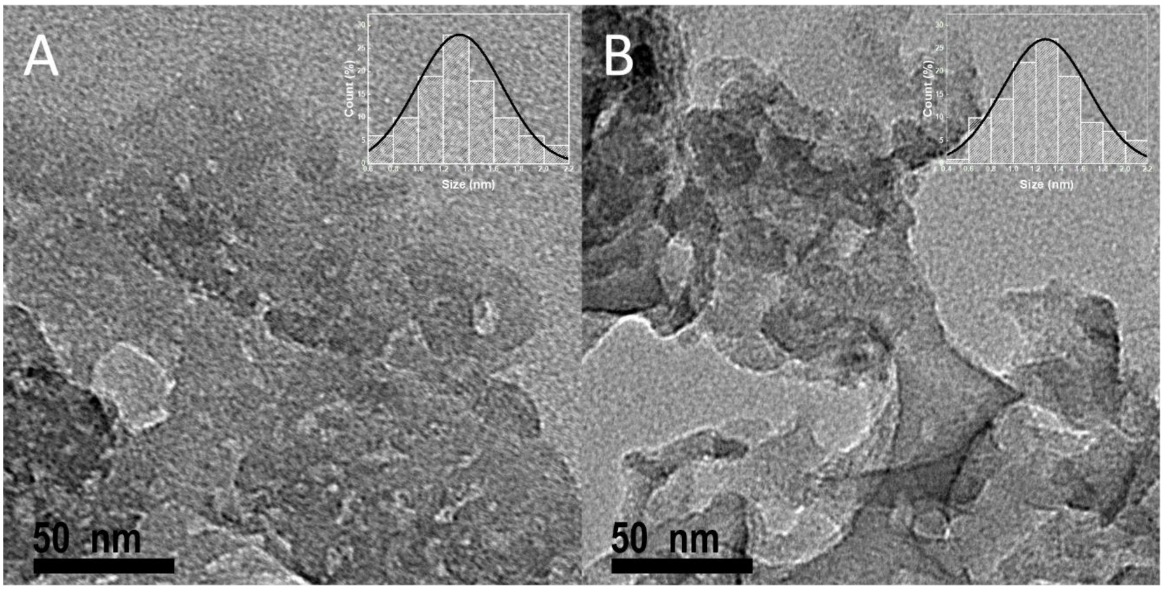

Figure S2. TEM images of Pt/CN50 (**A**) and Pt/CN100 (**B**). The inset represents the size distribution of Pt particles.

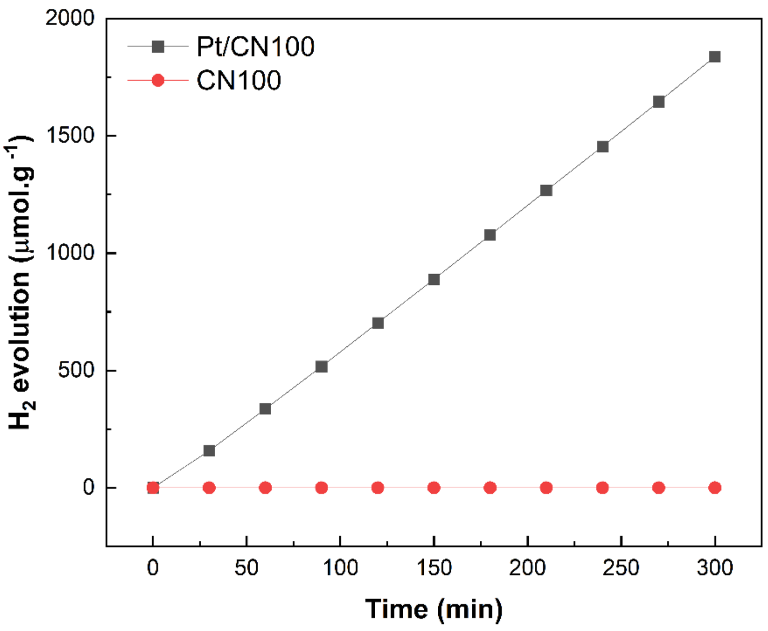

Figure S3. H_2 evolution results of Pt/CN100 and CN100 photocatalysts.

Table S1. The atomic percentages (at%) of COOH, C=O, and C-OH groups for Pt/CNx.

Sample	Pt/CN	Pt/CN0	Pt/CN50	Pt/CN100
COOH [a]	0	36.62	66.10	74.20
C=O [a]	100	37.03	26.88	18.15
C-OH [a]	0	26.35	7.02	7.65

[a] The numbers were obtained from the deconvolution of the N 1s XPS data.

Table S2. The atomic percentage ratios of Pt^0/Pt, Pt^{2+}/Pt, and Pt^{4+}/Pt for Pt/CNx.

Sample	Pt/CN0	Pt/CN50	Pt/CN100
Pt^0/Pt	0.08	0.17	0.14
Pt^{2+}/Pt	0.42	0.57	0.54
Pt^{4+}/Pt	0.50	0.25	0.32
